# Merits and Demerits of Machine Learning of Ferroelectric, Flexoelectric, and Electrolytic Properties of Ceramic Materials

**DOI:** 10.3390/ma17112512

**Published:** 2024-05-23

**Authors:** Kyuichi Yasui

**Affiliations:** National Institute of Advanced Industrial Science and Technology (AIST), Nagoya 463-8560, Japan; k.yasui@aist.go.jp

**Keywords:** machine learning, first principles calculations, PDE (partial differential equations) model, physical or phenomenological ODE (ordinary differential equations) model, predictive power, computational cost, experimental data, interpretability, beyond the parameter space of training data, all-dislocation-ceramics

## Abstract

In the present review, the merits and demerits of machine learning (ML) in materials science are discussed, compared with first principles calculations (PDE (partial differential equations) model) and physical or phenomenological ODE (ordinary differential equations) model calculations. ML is basically a fitting procedure of pre-existing (experimental) data as a function of various factors called descriptors. If excellent descriptors can be selected and the training data contain negligible error, the predictive power of a ML model is relatively high. However, it is currently very difficult for a ML model to predict experimental results beyond the parameter space of the training experimental data. For example, it is pointed out that all-dislocation-ceramics, which could be a new type of solid electrolyte filled with appropriate dislocations for high ionic conductivity without dendrite formation, could not be predicted by ML. The merits and demerits of first principles calculations and physical or phenomenological ODE model calculations are also discussed with some examples of the flexoelectric effect, dielectric constant, and ionic conductivity in solid electrolytes.

## 1. Introduction

Recently, data-driven science has been developed using machine learning (ML) [1,2,3]. ML is essentially a fitting procedure of many experimental data as a function of many factors called descriptors [2,4]. It has been argued that a lack of interpretability in ML could be a severe problem in terms of the reliability of the predicted values as well as development in scientific research based on causality [2,4,5,6,7,8]. On the other hand, the excellent fitting procedure of experimental data by means of ML could result in more accurate predicted values compared to first principles calculations, which could have some intrinsic systematic error [9]. Furthermore, ML could be computationally more economical compared to first principles calculations and could save time and effort [2,7,10,11]. ML could also save expensive and time-consuming experiments [12,13,14,15,16,17]. On the other hand, ML needs large amounts of pre-existing experimental data of high quality, which is not always possible [2,18,19]. Furthermore, prediction by ML is essentially restricted to the parameter range of pre-existing experimental data, and ML could not predict experimental results beyond the parameter space of the training data [20]. On the other hand, first principles calculations are entirely based on physical laws such as those of quantum mechanics [21,22,23,24]. The results of first principles calculations are basically interpretable, although it is sometimes difficult in practical terms due to their complex nature [24]. There is another category in computational science, namely simulations by a physical or phenomenological ODE (ordinary differential equations) model [24]. First principles calculations are mostly based on the partial differential equations (PDE) model [24]. Thus, ODE model calculations are usually computationally more economical and important factors are more easily traced compared to first principles calculations [24]. On the other hand, an ODE model is not fully based on first principles, and it needs validation by means of comparison with some experimental data or the results of first principles calculations [24]. In the present review, comparison between ML, first principles calculations (PDE models), and ODE model calculations is discussed from the viewpoint of their merits and demerits, especially in studies of ferroelectric, flexoelectric, and electrolytic properties of ceramic materials.

Ferroelectric materials have electric polarization even in the absence of an applied electric field, which is called spontaneous polarization [25,26,27,28,29,30]. As a result, the dielectric constant of ferroelectric materials is relatively high but strongly anisotropic [9,25,26,27,31,32]. For a single crystal of BaTiO_3_, the dielectric constant in the direction perpendicular to the spontaneous polarization is much higher than that parallel to spontaneous polarization [25,26,27]. On the other hand, for KDP (KH_2_PO_4_), the dielectric constant in the direction parallel to spontaneous polarization is considerably higher than that perpendicular to spontaneous polarization [25]. When the applied AC electric field is perpendicular to the spontaneous polarization, the dielectric response of ferroelectric material is due to the slight rotational vibration of the spontaneous polarization around the equilibrium position [33]. The dielectric constant (ε) at the frequency of the applied AC electric field is approximately the amplitude of temporal variation of polarization (${\left(\Delta P\right)}_{amp}$) in the direction of the applied AC electric field divided by the amplitude of the applied AC electric field ($E_{0}$) as follows [33,34]:

(1) $$\frac{\varepsilon }{\varepsilon_{0}}=1+\frac{1}{\varepsilon_{0}}\frac{\partial P}{\partial E}\approx \frac{{\left(\Delta P\right)}_{amp}}{\varepsilon_{0}E_{0}}$$

where $\varepsilon_{0}$ is the dielectric constant of a vacuum ($=8.854\times 10^{-12}$ F $m^{-1}$), $\varepsilon /\varepsilon_{0}$ is relative permittivity (or the dielectric constant), P is polarization in C $m^{-2}$, E is the applied electric field in V $m^{-1}$, ${\left(\Delta P\right)}_{amp}$ is in C $m^{-2}$, and $E_{0}$ is in V $m^{-1}$. Ferroelectric materials have been applied to multilayer ceramic capacitors (MLCCs), positive temperature-coefficient thermistors, ferroelectric memory devices, etc. [35,36].

**Figure 1:**
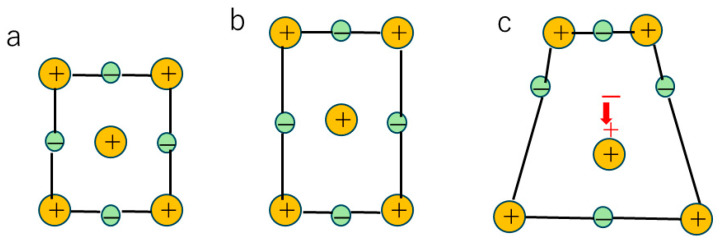
The flexoelectric effect. (**a**) no polarization without any strain. (**b**) no polarization with uniform strain. (**c**) flexoelectric polarization (indicated by the red arrow) with some strain gradient.

The flexoelectric effect is the appearance of electric polarization in any insulator crystals due to strain gradient (Figure 1) [33,37,38,39,40,41]. The piezoelectric effect, which is the appearance of electric polarization due to uniform strain, is only for non-centrosymmetric crystals [25]. On the other hand, the flexoelectric effect appears for any crystal symmetry [37,39,40]. Usually, the flexoelectric effect is negligibly small because of a relatively small flexoelectric coefficient and small strain gradient [37,38,39,40]. The flexoelectric coefficient is defined as follows [33,41,42]:

(2) $$P_{3}=\mu_{12}\frac{\partial \epsilon_{11}}{\partial x_{3}}$$

where $P_{3}$ is the flexoelectric polarization in C $m^{-2}$, $\mu_{12}$ is the flexoelectric coefficient in C $m^{-1}$, which is positive for BaTiO_3_ ($\mu_{12}\sim 10$ μC $m^{-1}$ at room temperature [41]), $\epsilon_{11}$ is the transverse strain, $x_{3}$ is the position in the thickness direction, and $\partial \epsilon_{11}/\partial x_{3}$ is the strain gradient in $m^{-1}$. Recently, the flexoelectric effect has been intensively studied because in nanomaterials the strain gradient could be large enough for a considerable flexoelectric effect [24,37,38,39,40,43,44,45,46]. The flexoelectric effect could be applied to energy harvesting technologies in nanoscale, which is the conversion of thermal, mechanical, and vibrational energy into electrical energy to power portable, wearable, or wireless electric devices without batteries [37,38,47,48,49,50,51,52,53].

In the present review, the application of ML to the electrolytic properties of materials is also discussed, especially the ionic conductivity of solid electrolytes. Recently, solid electrolytes have been intensively studied for application to all-solid-state batteries [54,55,56]. Lithium-ion batteries with flammable organic electrolytes have some safety problems because of their ignition capability due to overcharging or short-circuiting [55]. All-solid-state batteries are nonflammable and there is no liquid leakage, which could solve the safety issues of lithium-ion batteries [55]. Recently, the author has proposed that the ionic conductivity of solid electrolytes could be increased by several orders of magnitude without dendrite formation if solid electrolytes are filled with appropriate dislocations [57,58]. The high ionic conductivity along dislocations may be due to the lower formation energy of vacancies near dislocations [59,60,61,62]. Furthermore, fracture toughness, which is the resistance to crack propagation and is an important feature of engineering materials as it allows them to avoid failure, could be increased through the introduction of appropriate dislocations [58,63,64,65]. The higher fracture toughness of solid electrolytes could prevent dendrite formation and short-circuiting [58]. We call such a possible new type of solid electrolytes all-dislocation-ceramics [58,66]. We have also shown theoretically that all-dislocation-ceramics could be produced by dry pressing, which is sintering at relatively low temperatures under high applied pressure [66]. In the present review, it is pointed out that ML could not predict all-dislocation-ceramics as a candidate for materials with high ionic conductivity without dendrite formation, because ML hardly predicts experimental results beyond the parameter space of pre-existing experimental data.

## 2. Machine Learning (ML)

**Figure 2:**
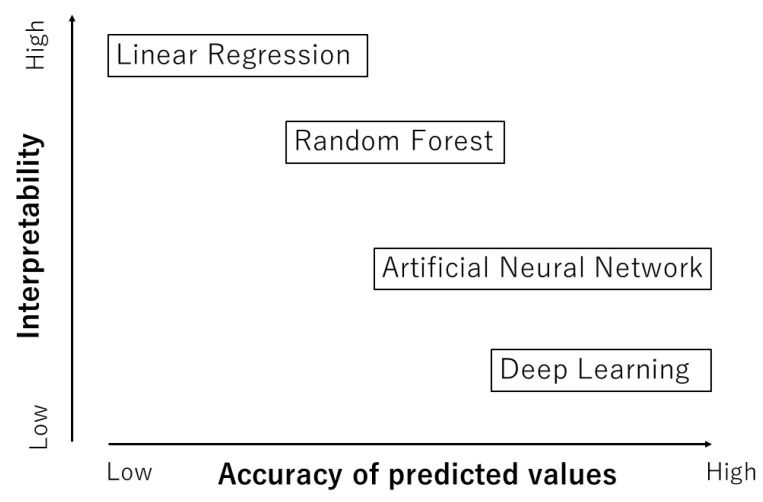
Machine learning (ML) models.

As already noted, ML is basically a fitting procedure of experimental data of some physical property such as the dielectric constant, ionic conductivity, etc., as a function of many factors called descriptors such as the diffusion coefficient, phase transition temperature, etc. [2,9,67]. After the fitting parameters are computationally determined using the training experimental data, a ML model with the determined parameters could predict the physical property under a given set of descriptors. This kind of ML model is called a regression model between the physical property and a set of descriptors [68]. There are a variety of ML models, from a highly interpretable one called a white-box model to a hardly interpretable one called a black-box model (Figure 2) [69]. A typical white-box model is linear regression which is a statistically and numerically determined linear relationship between a physical property and a set of descriptors [68,69]. In some cases, it is possible to predict a physical property by means of linear regression with a set of descriptors. Even in this case, the appropriate choice of the descriptors is essential for the linear regression to work effectively. For many other cases, however, linear regression is too simple to predict a physical property, and more sophisticated ML models need to be employed.

It has been reported that random forest (RF) works relatively well for many cases [70,71,72,73]. In RF, many decision trees are constructed by randomly resampling training data with randomly selected descriptors, where decision trees are regression models using regression trees branching off successively for different ranges of values of descriptors [69,74]. RF is the average of the outputs of many decision trees. A merit of RF is that the relative importance of descriptors in the regression model is numerically obtainable [69]. In other words, the interpretability of an RF model is relatively high (Figure 2).

**Figure 3:**
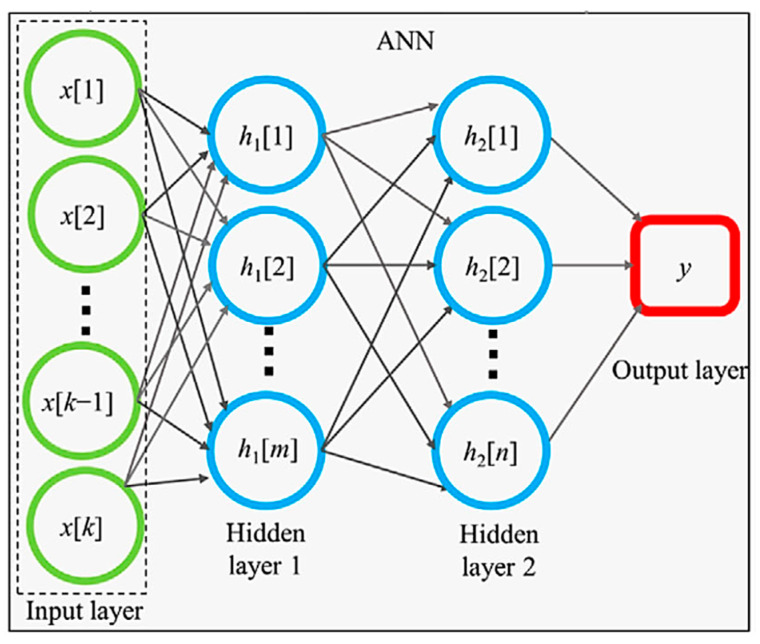
A model of an artificial neural network (ANN) with two hidden layers. Reprinted with permission from ref. [75]. Copyright 2020, Wiley.

In very complicated situations, RF is not sufficient for the fitting of the experimental data as a function of descriptors. In such cases, an artificial neural network (ANN) is often used [1]. ANN is based on a mathematical model developed to describe the activities of neurons in brains (Figure 3) [1,75]. In Figure 3, an ANN model consists of the input and output layers, and two hidden layers consisting of many mathematical neurons called formal neurons or artificial neurons, shown with blue circles. For each mathematical neuron, each input value ($x_{i}$) is multiplied by a weight ($w_{i}$) which is a fitting parameter, and they are summed up as follows.

(3) $$h=\sum_{i}w_{i}x_{i}$$

where h is the summed-up value and the summation is for all the input values to the neuron. The summed-up value (h) is nonlinearly transformed by using an activation function ($A\left(h\right)$) such as a step function (Equation (4)), sigmoid function (Equation (5)), rectified linear function (Equation (6)), etc., which is the output value from the mathematical neuron [1]. An activation function represents the firing of a biological neuron above the threshold strength of the input signal [1].

(4) $$\begin{array}{cc} y=A\left(h\right)=1 & \mathrm{for}\;h\geq 0\\ 0 & \mathrm{for}\;h<0 \end{array}$$

(5) $$y=A\left(h\right)=\frac{1}{1+e^{-h}}$$

(6) $$\begin{array}{cc} y=A\left(h\right)=h & \mathrm{for}\;h\geq 0\\ 0 & \mathrm{for}\;h<0 \end{array}$$

Each output value is used as a new input value for the next hidden layer (or the output layer), and the process is repeated. A set of weights ($w_{i}$) is different for a different neuron in the same or a different layer. In this way, an output value of an ANN model is numerically calculated (Figure 3) [1,75]. As an ANN model needs validation, the experimental data sets are divided into those for training and testing (by the ratio of 8:2, for example) [1,14]. Using the training sets of the experimental data, the fitting parameters are determined to reproduce the experimental data of a physical property as a function of various descriptors, which is the training process of an ANN model [1]. Then, the trained ANN model is tested by using the testing sets of the experimental data, which are often evaluated by the coefficient of determination ($R^{2}$ or *R*-squared) defined as in Equation (7) [76]:

(7) $$R^{2}=1-\frac{\sum_{i=1}^{M}{\left(F_{i}-Y_{i}\right)}^{2}}{\sum_{i=1}^{M}{\left(\bar{Y}-Y_{i}\right)}^{2}}$$

where $F_{i}$ is the value predicted by the trained ANN model under the *i*-th set of descriptors of the testing data, $Y_{i}\;$ is the corresponding experimental value, $\bar{Y}$ is the mean value of the testing experimental data, each summation is for all the testing sets of the experimental data, and M is the number of testing sets. The coefficient of determination is less than 1, and the best value for a trained ANN model is 1 [76]. When the coefficient of determination is close to 1, the ANN model is considered to have been validated. This process is called cross-validation.

Then, the trained ANN model could predict the physical property under a given set of descriptors. The accuracy of the values predicted by an ANN model is relatively high for many cases (Figure 2) [69]. However, if the training experimental data contain considerable error, the trained ANN model becomes inaccurate, for which it is said *garbage in*, *garbage out* [77]. The interpretability of an ANN model is relatively low because an ANN model is very complex from the viewpoint of simple causality [78]. Thus, it is called a black-box model [69]. However, there have been some reports of improvements in the interpretability of an ANN model [2,8,69,79,80,81].

**Figure 4:**
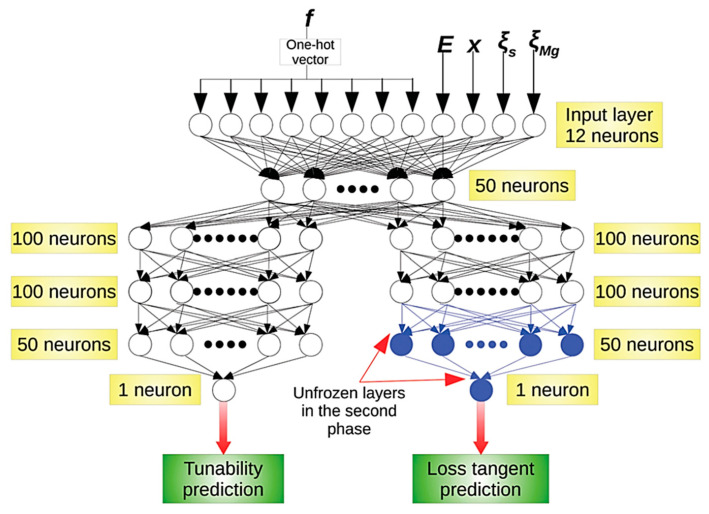
A deep neural network (deep learning) model with four hidden layers. In this example, a DL model was first trained with the results of theoretical calculations on tunability, which is the magnitude of the decrease in the dielectric constant divided by the initial dielectric constant, and the loss tangent which is the dielectric loss, under a given frequency (*f*), electric field (*E*), barium proportion (*x*) in MgO-doped BST (Ba_x_Sr_1-x_TiO_3_), the defect factor ($\xi_{s}$), and that due to MgO ($\xi_{Mg}$). Then, the DL model was trained with the experimental data only for the loss tangent for the unfrozen layers. Reprinted with permission from ref. [82]. Copyright 2020, The Royal Society of Chemistry.

When the number of the hidden layers in ANN is relatively large (usually more than four, sometimes more than two), it is called deep learning (DL) (Figure 4) [69,82]. The *expressive power* of ANN dramatically increases as the number of the hidden layers increases (even if the increase is only one) [83,84,85,86,87]. The expressive power increases much more by depth (the number of the hidden layers) than by width (the number of neurons per layer; *m* or *n* in Figure 3) [83]. It has been reported that the total number of neurons needed by a shallow network to approximate a smooth function is exponentially larger than the corresponding number of neurons needed by a deep network [87]. It should be noted that there should be an upper bound for the total number of neurons in hidden layers in order to prevent the network from overfitting the data [82,88]. This upper bound is determined by the amount of training data available and the number of input and output neurons [82,88]. There is no specific rule for the number of hidden layers or number of neurons in each layer, which are usually determined by intuition or trial-and-error [82].

DL works quite well especially for the classification of images such as photographs, hand-written letters and numbers, etc. [89]. A DL model could be trained by many such images which are converted into numerous numbers indicating the brightness and color of each pixel in each image [89]. The typical number of pixels in each image is 256 × 256 or more [89]. A DL model can be used to predict a physical property under a given image of electron microscopy of a sample [14,15,90]. Convolutional neural networks (CNNs), which are a special kind of ANN (DL), work especially effectively in the analysis of images [14,15,90]. However, the mechanism for the excellent effectiveness of DL in the analysis of images has not yet been fully understood [83,89].

ML is not restricted to the prediction of a single physical property, but is also applicable in the prediction of multiple physical properties as a function of descriptors [91]. It has been suggested that a simpler (interpretable) ML model would be better if the accuracy of predicted values for pre-existing data is sufficiently high from the point of view of the predictive power for wider ranges of descriptors [89]. With regard to the computational time needed to train a ML model, a simpler ML model such as RF often requires a relatively short time such as a few minutes, but a DL model sometimes requires much more time, such as 6 h as in the case of Figure 4 [82]. In DL, higher number of hidden layers results in much slower training [82]. ML is a still-developing field of research in many fields of science and engineering [69,89].

## 3. First Principles Calculations (PDE Models)

### 3.1. What Are First Principles Calculations?

First principles calculations are based on fundamental physical laws, mostly without using free parameters [22]. Examples are DFT (density functional theory) calculations for electron density and the ground-state energy based on principles of quantum mechanics [21,22,92], computational fluid dynamics (CFD) simulations based on the fundamental equations of fluid dynamics [93], finite element method (FEM) calculations applied to the mechanics of materials [94,95], molecular dynamics simulations [96,97], etc. In the present section, DFT calculations of electron density and the first principles calculations of flexoelectric coefficients are discussed.

### 3.2. DFT (Density Functional Theory)

As the mass of an atomic nucleus is significantly larger than that of an electron, the position of the atomic nucleus is fixed, and only the electronic wave function is numerically calculated by solving the Schrödinger equation [21]. However, direct numerical solution of the following Schrödinger equation for a multielectron system (Equation (8)) is practically impossible when the number of electrons is relatively large [21]:

(8) $${\hat{H}}_{elec}\Psi \left(\vec{r_{1}},\cdots ,\vec{r_{N}}\right)=E\Psi \left(\vec{r_{1}},\cdots ,\vec{r_{N}}\right)$$

(9) $${\hat{H}}_{elec}=\sum_{i}\left[\frac{{\hat{p}}_{i}^{2}}{2m_{e}}+V_{ext}\left(\vec{r_{i}}\right)\right]+\frac{1}{2}\sum_{ij}\frac{e^{2}}{\left|\vec{r_{i}}-\vec{r_{j}}\right|}$$

(10) $$V_{ext}\left(\vec{r}\right)=\sum_{I}\frac{e^{2}Z_{I}}{\left|\vec{r}-\vec{R_{I}}\right|}$$

where ${\hat{H}}_{elec}$ is the electron’s Hamiltonian operator (the hat means operator), $\Psi \left(\vec{r_{1}},\cdots ,\vec{r_{N}}\right)$ is a multielectron wavefunction of N electrons with the position vectors of $\vec{r_{1}},\cdots ,\vec{r_{N}}$, E is an energy eigenvalue, ${\hat{p}}_{i}$ is momentum operator of the *i*-th electron as ${\hat{p}}_{i}=-i\hbar \nabla_{i}$ where i is the imaginary unit (while the subscript i indicates the *i*-th electron), $\hbar =\frac{h}{2\pi }$, h is the Planck constant (=$6.626\times 10^{-34}$ J s), $\nabla_{i}=\left(\frac{\partial }{\partial x_{i}},\frac{\partial }{\partial y_{i}},\frac{\partial }{\partial z_{i}}\right)$, ${\hat{p}}_{i}^{2}=-\hbar^{2}\nabla_{i}^{2}$, $\nabla_{i}^{2}=\frac{\partial^{2}}{\partial x_{i}^{2}}+\frac{\partial^{2}}{\partial y_{i}^{2}}+\frac{\partial^{2}}{\partial z_{i}^{2}}$, $m_{e}$ is the electron mass (=$9.109\times 10^{-31}$ kg), $V_{ext}\left(\vec{r_{i}}\right)$ is the potential energy of the *i*-th electron in the Coulomb fields of atomic nuclei, I indicates the *I*-th atomic nucleus with electric charge of $eZ_{I}$ and position vector $\vec{R_{I}}$, the summation in Equation (10) is for all the atomic nuclei, and the last term on the right side of Equation (9) is the interaction energy between electrons [21]. Due to the complex nature of the interaction energy between electrons, the Schrödinger equation (Equation (8)) can hardly be solved even numerically [21].

In order to solve the problem, DFT is used, in which a multielectron system is described by a single electron system using a mean potential as follows [21]:

(11) $${\hat{H}}_{KS}\psi_{j}\left(\vec{r}\right)=E_{j}\psi_{j}\left(\vec{r}\right)$$

(12) $$n\left(\vec{r}\right)=\sum_{j=1}^{N}{\left|\psi_{j}\left(\vec{r}\right)\right|}^{2}$$

(13) $${\hat{H}}_{KS}=\frac{{\hat{p}}^{2}}{2m_{e}}+V_{KS}^{\left[n\right]}\left(\vec{r}\right)$$

(14) $$V_{KS}^{\left[n\right]}\left(\vec{r}\right)=V_{ext}\left(\vec{r}\right)+V_{H}^{\left[n\right]}\left(\vec{r}\right)+V_{XC}^{\left[n\right]}\left(\vec{r}\right)$$

(15) $$V_{H}^{\left[n\right]}\left(\vec{r}\right)=e^{2}\int \frac{n\left(\vec{\overset{´}{r}}\right)}{\left|\vec{r}-\vec{\overset{´}{r}}\right|}d^{3}\overset{´}{r}$$

(16) $$V_{XC}^{\left[n\right]}\left(\vec{r}\right)=\frac{\delta E_{XC}\left[n\right]}{\delta n\left(\vec{r}\right)}$$

where ${\hat{H}}_{KS}$ is the effective Hamiltonian operator for a single electron system called the Kohn–Sham Hamiltonian, $\psi_{j}\left(\vec{r}\right)$ is a single electron wavefunction with position vector $\vec{r}$ and is the *j*-th eigen function of the Kohn–Sham Hamiltonian, $E_{j}$ is the *j*-th eigen energy, $n\left(\vec{r}\right)$ is the electron density as a function of position ($\vec{r}$), the summation on the right side of Equation (12) is for the set of *N* eigen functions with the lowest value of the sum of N eigen energies, $\hat{p}=-i\hbar \nabla$, $\nabla =\left(\frac{\partial }{\partial x},\frac{\partial }{\partial y},\frac{\partial }{\partial z}\right)$, $V_{KS}^{\left[n\right]}$ is called the Kohn–Sham potential and the superscript [*n*] means that it is a functional of the electron density ($n\left(\vec{r}\right)$), $V_{ext}\left(\vec{r}\right)$ is given by Equation (10), $V_{H}^{\left[n\right]}\left(\vec{r}\right)$ is the potential due to Coulomb repulsion from the electron cloud which is a functional of electron density ($n\left(\vec{r}\right)$) and is called Hartree potential, $V_{XC}^{\left[n\right]}\left(\vec{r}\right)$ is the potential due to the exchange–correlation interaction of electrons caused by the Pauli exclusion principle of quantum mechanics for electrons with parallel spins (exchange interaction) and by nonclassical Coulomb repulsion for electrons with antiparallel spins (correlation interaction) [21,92,98,99,100], $E_{XC}[n]$ is the exchange–correlation energy, and the right side of Equation (16) is the functional derivative of $E_{XC}[n]$ [21]. The initial procedure is to guess electron density ($n\left(\vec{r}\right)$) and construct the Kohn–Sham Hamiltonian [92]. Then, solving the Schrödinger-like equation (Equation (11); Kohn–Sham equation), the newly derived electron density ($n\left(\vec{r}\right)$ in Equation (12)) is inserted back into Equations (15) and (16) to obtain a new Kohn–Sham Hamiltonian. This process is repeated until a self-consistent solution for $n\left(\vec{r}\right)$ and $V_{KS}\left(\vec{r}\right)$ is obtained [21]. As a result, the ground state and other properties of a multielectron system can be determined from the knowledge of electron density ($n\left(\vec{r}\right)$) because $V_{H}^{\left[n\right]}\left(\vec{r}\right)$ and $V_{XC}^{\left[n\right]}\left(\vec{r}\right)$ are functionals of electron density. Thus, the theory is called the density functional theory (DFT). As ${\hat{p}}^{2}=-\hbar^{2}\nabla^{2}$, the Schrödinger-like equation (Equation (11)) is a PDE (partial differential equation).

### 3.3. DFT Calculations

**Figure 5:**
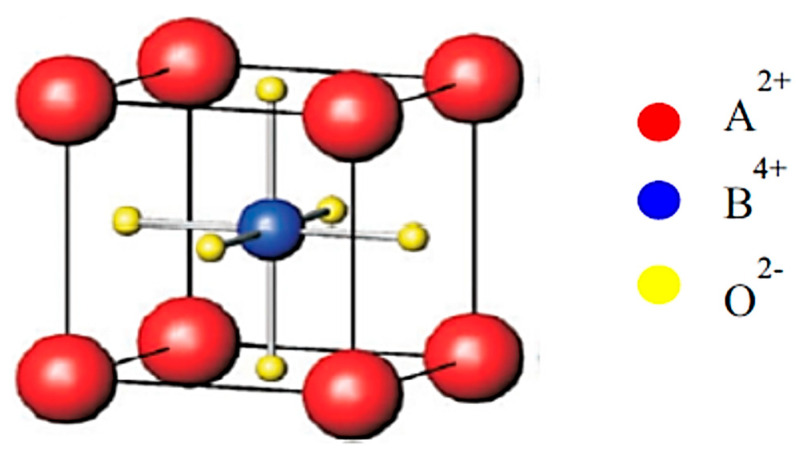
The perovskite structure. Reprinted with permission from ref. [101]. Copyright 2019, Elsevier.

**Figure 6:**
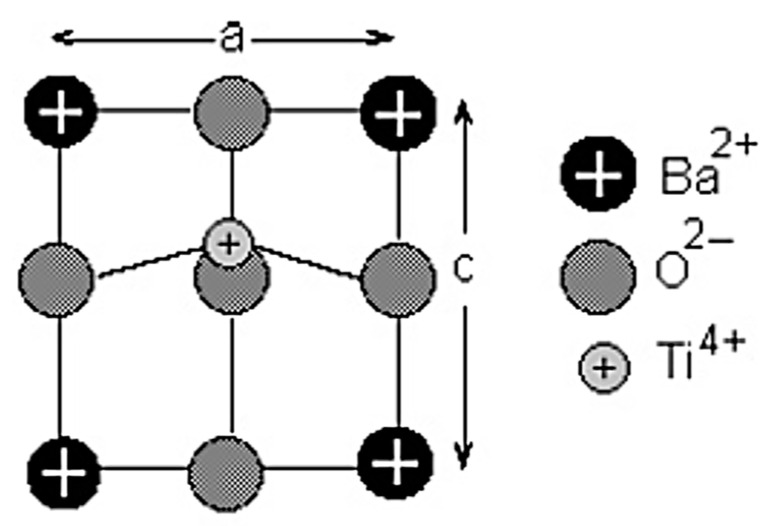
The tetragonal unit cell of BaTiO_3_. Reprinted with permission from ref. [102]. Copyright 2003, Springer Nature.

Barium titanate (BaTiO_3_) is one of the most important ferroelectric materials, and BaTiO_3_-based materials have been used in MLCCs, etc. [25,26,27,35,36]. BaTiO_3_ has a perovskite structure as shown in Figure 5 [101]. The perovskite structure is seen for crystals with the chemical formula of ABX_3_ where A and B are two different cations, and X is an anion that bonds to both A and B [101]. X is often oxygen, and it is called perovskite oxides, denoted by ABO_3_ [26,27,101]. Perovskite oxides have a cubic or nearly cubic crystal structure (Figure 5) [101]. For BaTiO_3_, A=Ba and B=Ti. At room temperature, the Ti atom (positive ion) is slightly displaced from the center of a cube, and accordingly there is electric polarization even without an applied electric field called spontaneous polarization, which is the origin of the ferroelectricity in BaTiO_3_ (Figure 6) [102]. The experimental lattice constants for BaTiO_3_ in Figure 6 are a=3.9945 Å and c=4.0335 Å. The atomic positions in units of a along the X and Y axis and c along the Z axis are as follows: Ba at (0,0,0), Ti at (0.5,0.5,0.514), O_1_ at (0.5,0.5,−0.025), and O_2_ at (0,0.5,0.488) and (0.5,0,0.488) (Figure 6) [102,103,104,105].

**Figure 7:**
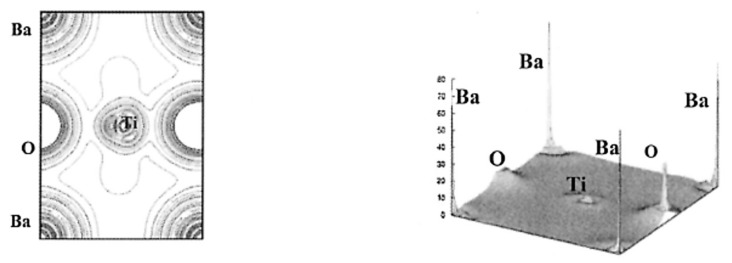
The results of the first principles calculations (DFT calculations) on the electron density in the (110) plane of BaTiO_3_ (**left**) and in three dimensions (**right**). Reprinted with permission from ref. [102]. Copyright 2003, Springer Nature.

**Figure 8:**
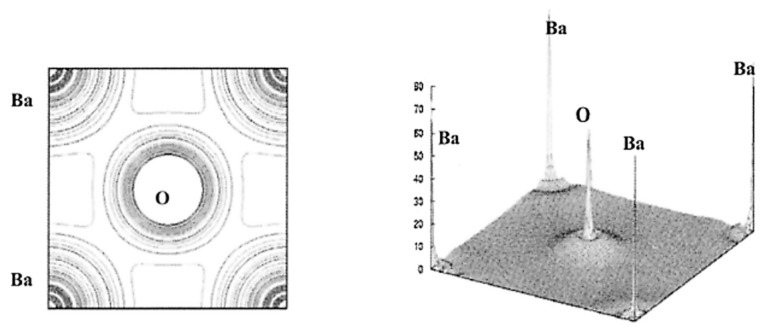
The results of the first principles calculations (DFT calculations) on the electron density in the (100) plane of BaTiO_3_ (**left**) and in three dimensions (**right**). Reprinted with permission from ref. [102]. Copyright 2003, Springer Nature.

The results of the DFT calculations for BaTiO_3_ on the spatial distribution of electron density ($n\left(\vec{r}\right)$) are shown in Figure 7 and Figure 8 [102]. From Figure 7, the Ti-O bond has covalent character as there is noticeable charge distribution in the middle of the Ti-O bond. On the other hand, the Ba-O bond is typically ionic because there is not much bonding charge and the charge density distribution around Ba is almost spherically symmetric (Figure 8) [102]. According to the charge distribution, the size of Ba atoms is much larger than that of Ti and O. The size of O atoms is slightly larger than that of Ti atoms. The atomic numbers of Ba, Ti, and O are 56, 22, and 8, respectively. The calculated value of the band gap of 2.3 eV is slightly smaller than the experimental values of about 3 eV [102,106]. The reason for the discrepancy may be due to the generalized gradient approximation (GGA) used in the derivation of the exchange–correlation functional ($E_{XC}[n]$) [21,102,107,108]. In DFT calculations, some approximation needs to be used in the derivation of the exchange–correlation functional ($E_{XC}[n]$), which is a central problem in the DFT formalism [21]. In other words, some inevitable systematic error may occur even in the first principles calculations (DFT calculations). Furthermore, impurities and defects such as vacancies and dislocations are often neglected in the first principles calculations, which also causes some discrepancy between the calculated results and experimental data [44,109]. DFT calculations are generally restricted to zero temperature (the “athermal limit”) because it is computationally very expensive to treat temperature, which could also be another reason for the discrepancy [110,111]. In order to consider the effect of temperature, ab initio molecular dynamics simulations are often performed, in which the forces between atoms (molecules) are calculated with a DFT-based first principles calculation at each time-step and motions of atoms (molecules) are calculated by Newton’s classical equation of motion [96,110].

With regard to the computational time of DFT-based calculations, it ranges from about 1 h for NMR chemical shift calculations [112] to about 250 h for finding out the right crystal structure of compounds with CPU core of Intel(R) Xeon(R) Silver 4210 CPU @ 2.20 GHz [113]. The computational time for DFT-based calculations could be significantly longer than that for ML and ODE model calculations.

### 3.4. Flexoelectric Effect

In general, the flexoelectric coefficient is expressed as a flexoelectric tensor in $C\;m^{-1}$($\mu_{ijkl}$, where the subscript i,j,k,l are the numbers 1, 2, or 3 corresponding to x, y, z, respectively) as follows [33,37,38,39]:

(17) $$P_{l}=\sum_{ijk}\mu_{ijkl}\frac{\partial \epsilon_{ij}}{\partial x_{k}}$$

where $P_{l}$ is flexoelectric polarization in $C\;m^{-2}$, $\epsilon_{ij}$ is the strain tensor, and $x_{k}$ is the position coordinate in m.

According to the first principles theory by Hong and Vanderbilt [114], the flexoelectric tensor is calculated by means of the following equations:

(18) $$\mu_{ijkl}=\mu_{ijkl}^{ld}+\mu_{ijkl}^{lq}+\mu_{ijkl}^{el}$$

where the terms on the right side of Equation (18) are the lattice dipole, lattice quadrupole, and electronic terms, respectively. They are calculated as follows [114]:

(19) $$\mu_{ijkl}^{ld}=V_{c}^{-1}\sum_{I\tau }Q_{Ii\tau }^{\left(1\right)}N_{I\tau jkl}$$

(20) $$\mu_{ijkl}^{lq}=-\frac{1}{4}V_{c}^{-1}\sum_{I\tau }\left(Q_{Ii\tau l}^{\left(2\right)}\Gamma_{I\tau jk}+Q_{Ii\tau k}^{\left(2\right)}\Gamma_{I\tau jl}\right)+\mu_{ijkl}^{lq,J}$$

(21) $$\mu_{ijkl}^{el}=\frac{1}{6}V_{c}^{-1}\sum_{I}Q_{Iijkl}^{\left(3\right)}+\mu_{ijkl}^{el,J}$$

(22) $$Q_{Ii\tau }^{\left(1\right)}=\int r_{i}f_{I\tau }\left(\vec{r}\right)d^{3}r\;$$

(23) $$Q_{Ii\tau j}^{\left(2\right)}=\int r_{i}f_{I\tau }\left(\vec{r}\right)r_{j}d^{3}r$$

(24) $$Q_{Ii\tau jk}^{\left(3\right)}=\int r_{i}f_{I\tau }\left(\vec{r}\right)r_{j}r_{k}d^{3}r$$

where $V_{c}$ is the considered volume containing several cells in $m^{3}$, $Q_{Ii\tau }^{\left(1\right)},\;Q_{Ii\tau j}^{\left(2\right)},\;Q_{Ii\tau jk}^{\left(3\right)}$ are moments of the induced charge redistribution in C, C m, $C\;m^{2}$, respectively, and $\Gamma_{I\tau jk}$ and $N_{I\tau jkl}$ are in m and $m^{2}$, respectively, and defined as follows [114]:

(25) $${\vec{u}}_{lI}=\left({\vec{u}}^{\left(0\right)}+{\vec{u}}_{I}^{\left(1\right)}+{\vec{u}}_{I}^{\left(2\right)}\right)e^{i\vec{k}\cdot {\vec{R}}_{lI}}$$

(26) $$u_{I\tau }^{\left(1\right)}=\sum_{jk}\Gamma_{I\tau jk}\eta_{jk}$$

(27) $$u_{I\tau }^{\left(2\right)}=\sum_{jkl}N_{I\tau jkl}\nu_{jkl}$$

where ${\vec{u}}_{lI}$ is the total displacement of atom *I* in cell *l* in m, ${\vec{u}}^{\left(0\right)}$ is the “acoustic” displacement of the cell as a whole (independent of atom index *I*) in m, ${\vec{u}}_{I}^{\left(1\right)}$ is the additional displacement in m induced by strain tensor $\eta_{jk}$, ${\vec{u}}_{I}^{\left(2\right)}$ is the additional displacement in m induced by strain gradient tensor $\nu_{jkl}$ which is in $m^{-1}$, $\vec{k}$ is the wave vector of a long-wavelength displacement wave in $m^{-1}$ (“frozen acoustic phonon”) considered in the crystal, ${\vec{R}}_{lI}$ is the initial position vector of atom *I* in cell *l* in m, $u_{I\tau }^{\left(1\right)}$ and $u_{I\tau }^{\left(2\right)}$ are the τ-components of the vectors ${\vec{u}}_{I}^{\left(1\right)}$ and ${\vec{u}}_{I}^{\left(2\right)}$, respectively, $\Gamma_{I\tau jk}$ is the internal-strain tensor describing the additional atomic displacement induced by a strain, and $N_{I\tau jkl}$ is the corresponding tensor describing the response to a strain gradient. In Equations (20) and (21), the last terms on the right side are extra anti-symmetric contributions [113]. In Equations (22)–(24), $r_{1}=x,\;r_{2}=y,\;r_{3}=z$, and $f_{I\tau }\left(\vec{r}\right)$ is in $C\;m^{-4}$ and is defined as follows [114].

(28) $$f_{I\tau }\left(\vec{r}-{\vec{R}}_{lI}\right)=\frac{\partial n\left(\vec{r}\right)}{\partial u_{lI\tau }}$$

where the right side means the change in charge density ($n\left(\vec{r}\right)$ in $C\;m^{-3}$) induced by the displacement of atom *I* in cell *l* initially at ${\vec{R}}_{lI}$, by a distance $u_{lI\tau }$ along direction τ, keeping all other atoms fixed.

The flexoelectric tensor given by Equation (18) is computed in the context of DFT calculations [114]. The first principles calculations of the flexoelectric coefficients yield the values in the order of nC $m^{-1}$ with a negative sign, which strongly disagree with the experimental values on the order of μC m^−1^ with a positive sign [41,114,115,116,117,118,119,120,121]. One of the reasons for the disagreement may be due to the neglect of the surface effect in the first principles calculations [114,122,123,124]. It may be concluded, however, that first principles calculations do not always reproduce experimental data and that the calculated results may even strongly disagree with the experimental data. On the other hand, ML is basically a fitting procedure of experimental data and could reproduce experimental data relatively well for many cases within the range of the training data.

## 4. ML Supporting and/or Accelerating First Principles Calculations

ML has also been used to support or accelerate first principles calculations [2]. One such application is to find density functionals for DFT calculations with ML [2,125,126,127,128,129,130]. Tozer et al. [125] employed the Zhao–Morrison–Parr (ZMP) approach for the determination of the exchange–correlation potentials of molecules by using charge density $n\left(\vec{r}\right)$, by which a substantial amount of near-exact data in the form of $n\left(\vec{r_{i}}\right),\;\nabla n\left(\vec{r_{i}}\right),\;\nabla \nabla n\left(\vec{r_{i}}\right),\;\cdots ,\;V_{XC}\left(\vec{r_{i}}\right)$ is obtained, where $\vec{r_{i}}$ refers to a grid point in a certain molecule. In conventional DFT calculations, a value for the exchange–correlation potential at a point in space is obtained from an analytic expression using a knowledge of the charge density $n\left(\vec{r}\right)$ and possibly its gradient at that point. With the large amount of data obtained by the ZMP approach for many molecules, it may be possible to form an approximate potential to some function of the density and its derivatives. Tozer et al. [125] have investigated how well a fit can be achieved using an artificial neural network (ANN) rather than choosing an explicit functional form. Using ANN with only one hidden layer, they obtained successful results [125].

Nagai et al. [126] trained an ANN model with two hidden layers to produce the projection from the charge density $n\left(\vec{r}\right)$ onto the exchange–correlation potential $V_{xc}\left(\vec{r}\right)$. They successfully replaced a procedure in DFT calculations with the ML projection [126]. There are also several other reports on the usage of ML in obtaining approximate density functionals [127,128,129,130].

Another application of ML to support and/or accelerate first principles calculations is the fast estimation of an interatomic force field using ML to accelerate molecular dynamics (MD) simulations [2,51,131,132,133,134,135]. Compared to DFT calculations, which provide microscopic insight into the electronic structure of ferroelectrics [92], MD simulations are advantageous for investigating the macroscopic properties of ferroelectrics [133] such as dielectric response [136], electromechanical response [137], phase transition [138], and the growth of domain walls [139]. However, a realistic MD simulation is extremely difficult because accurate force field calculations by means of DFT are computationally very expensive [2,133]. In order to solve this problem, the estimation of an interatomic force field via ML is sometimes used because it is computationally more economical [2,133]. Thong et al. [133] reported that the results of MD simulations based on ML interatomic potential sufficiently agree with the results of DFT calculations of the atomic force, elastic properties, and phonon dispersion of the ferroelectric KNbO_3_ perovskite. Wang et al. [134] reported that the dynamical nature of ferroelectric phase transition between the rhombohedral and cubic structures of GeTe is successfully reproduced by MD simulations using ML interatomic potentials. Wu et al. [135] reported that the structural properties of ferroelectric HfO_2_ such as elastic constants, phonon dispersion relationship, and phase transition barriers predicted by MD simulations using an ML interatomic force field agree with the results of DFT calculations. ML interatomic potentials have also been successfully used in MD simulations of the flexoelectric effect in 2D van der Waals bilayers, as reported by Javvaji et al. [51].

There are several other applications of ML to accelerate (approximate) first principles calculations. Rahman et al. [11] reported that an ANN model trained by the total energies of BaTiO_3_ with random lattice parameters calculated by DFT successfully predicts the ground states at various misfit strains of epitaxially strained BaTiO_3_. In other words, ML could accelerate (approximate) first principles calculations to search for the polar phase stability of ferroelectric oxide. Alhada-Lahbabi et al. [140] reported that ML could accelerate (approximate) ferroelectric phase-field modeling. Jalem et al. [141] reported that Bayesian-driven first principles calculations could accelerate exploration of fast ion conductors for rechargeable battery application. Bayesian optimization is the statistical prediction of optimum condition by finding the maximum value of an acquisition function which could be the probability of improvement, expected improvement, upper or lower confidence bound, etc. [142,143]. In summary, the application of ML to accelerate and/or support first principles calculations could be a promising field of research. It is another kind of application of ML compared to a fitting procedure of experimental data to predict experimental results.

## 5. Physical or Phenomenological ODE Models

### 5.1. What Is an ODE Model?

In many ODE models, the independent variable is time, and physical quantities such as density, mobile and immobile dislocation densities, etc. are assumed to be spatially uniform [24,66,144,145,146]. As a result, equations describing some physical phenomena could contain only derivatives with respect to the single variable (time), which are ordinary differential equations (ODEs). Numerical simulations with an ODE model are computationally much more economical compared to first principles calculations [24]. Furthermore, it is much easier to trace important factors and to perform numerical simulations for a wide range of parameters compared to first principles calculations [24]. In many cases, the purpose of numerical computations is not numbers but insight [93,147]. For this purpose, numerical simulations with an ODE model are sometimes superior to first principles calculations [24]. The author has recognized the merits of ODE modeling through the studies of chemical reactions inside cavitation bubbles under ultrasound called sonochemical reactions [24,148,149]. In an ODE model of bubble dynamics, temperature and pressure are assumed to be spatially uniform inside a bubble, and the independent variable is time [24,148,149]. In studies of sonochemistry and sonoluminescence, which is the light emission phenomena from cavitation bubbles under ultrasound [150,151,152,153], an ODE model of bubble dynamics could sufficiently reproduce the experimental results [154,155]. From such studies, many mechanisms of sonochemistry and sonoluminescence have been clarified [148,149]. In the present section, an ODE model of the flexoelectric effect is discussed.

### 5.2. Flexoelectric Effect

In the present subsection, an ODE model for the dynamic response of the flexoelectric polarization is discussed [33,43]. The motivation is to study the mechanism for the experimental results of a high dielectric constant with nearly flat temperature dependence for the ordered assemblies of BaTiO_3_ nanocubes [156,157]. In the experiments, the ordered assembly was fabricated by dip coating with a very slow withdrawal speed from mesitylene solution in which 15 nm BaTiO_3_ nanocubes capped with oleic acid were dispersed. The oriented attachment of BaTiO_3_ nanocubes may be due to electric dipole–dipole interaction between nanocubes originating in spontaneous polarization of BaTiO_3_ [158,159]. The ordered assembly was calcinated at 400 °C for 1 h and sintered at 850 °C for 1 h in O_2_ [156]. The ordered structure, in which nanocubes were attached face to face similarly to building blocks, was maintained even after the sintering, with the size and shape of each nanocube remaining unchanged [156,157]. The top electrodes were fabricated on the ordered assembly by means of electron beam deposition with a mask with the hole diameter of 10 μm. The dielectric constant of the assembly was measured to be about 2600 and 3800 at 1 MHz at room temperature for samples of 580 and 290 nm in thickness, respectively [156,157]. For both thicknesses, the amplitude of the applied AC voltage was 0.5 V. Thus, the amplitude of the AC field was 8.62 kV $cm^{-1}$ and 17.24 kV $cm^{-1}$ for thicknesses of 580 and 290 nm, respectively. The measured dielectric constant is much higher than that of typical BaTiO_3_ thin films (which is lower than 1000) [33,43,160,161].

**Figure 9:**
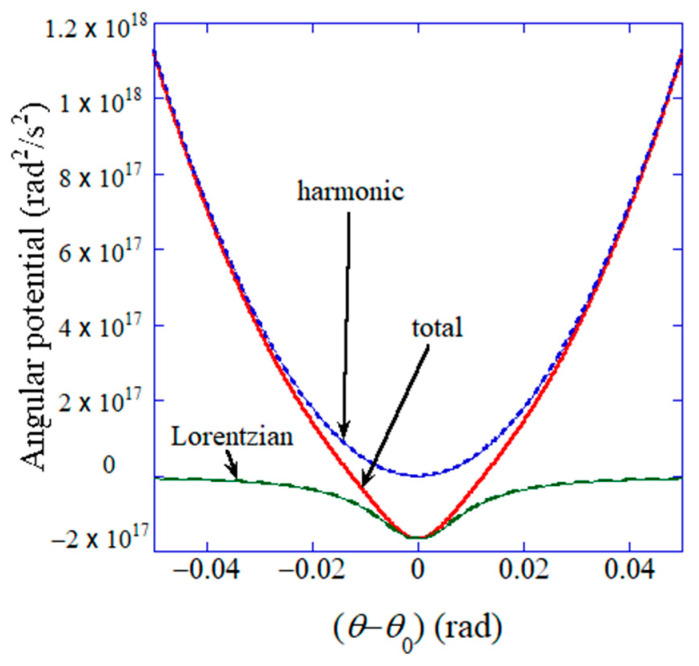
Assumed angular potential for the dynamic response of flexoelectric polarization. θ is the angle of flexoelectric polarization relative to the x-axis, and $\theta_{0}$ is the equilibrium angle of flexoelectric polarization ($\theta_{0}=0$ is assumed). Reprinted with permission from ref. [43]. Copyright 2022, MDPI.

In order to study the mechanism of the high dielectric constant of the ordered assemblies of BaTiO_3_ nanocubes, the strain gradient inside a nanocube caused by a small tilt angle between two attached nanocubes is estimated as $2.7\times 10^{5}$ $m^{-1}$ [43]. Then, from Equation (2), the magnitude of flexoelectric polarization is estimated as 2.7 C $m^{-2}$, which is one order of magnitude larger than that of the spontaneous polarization of BaTiO_3_ [43]. When the applied AC field is parallel to the flexoelectric polarization, however, the flexoelectric polarization could not respond to the applied AC field because some mismatch of strain should occur at the interface between the two attached nanocubes [43]. Instead, some ferroelectric component would contribute to the dielectric constant [43]. On the other hand, the flexoelectric polarization perpendicular to the applied AC field contributes to the dielectric constant [43]. In the ODE model [43], the dynamic response of the flexoelectric polarization is simply modeled by the following classical equation of rotational motion for the electric dipole:

(29) $$I\frac{d^{2}\theta }{dt^{2}}=\left(p\cos\theta \right)E_{0}\sin\left(\omega_{E}\right)-k\left(\theta -\theta_{0}\right)-\frac{\beta \left(\theta -\theta_{0}\right)}{{\left(1+\zeta {\left(\theta -\theta_{0}\right)}^{2}\right)}^{2}}-\lambda \frac{d\theta }{dt}$$

where I is the (virtual) moment of inertia in kg $m^{2}\;{\left(rad\right)}^{-1}$ for the electric dipole, θ is the angle of polarization relative to *x*-axis in rad, t is time in s, p is the (virtual) electric dipole moment in C m, which is proportional to flexoelectric polarization (P in C $m^{-2}$) as p=PV, V is volume in $m^{3}$, $\omega_{E}\;$ is the angular frequency in $s^{-1}\;$ of the applied electric field, k is the spring constant in N m ${\left(rad\right)}^{-1}$ for angular harmonic potential, $\theta_{0}$ is the equilibrium angle of polarization in rad, β and ζ are coefficients for angular Lorentzian potential (β is in N m ${\left(rad\right)}^{-1}$ and ζ is in ${\left(rad\right)}^{-2}$), and λ is the angular damping constant in J s ${\left(rad\right)}^{-1}$. The component of polarization parallel to the applied electric field (*y* direction) is $P_{y}=\left|P\right|\sin\theta$, by which ${\left(\Delta P\right)}_{amp}$ in Equation (1) is determined and the dielectric constant is obtained. In Equation (29), the following nonlinear angular potential ($U_{\theta }$) is used [43]:

(30) $$U_{\theta }=\frac{k}{2I}{\left(\theta -\theta_{0}\right)}^{2}-\frac{\beta }{2\zeta I}\times \frac{1}{\left(1+\zeta {\left(\theta -\theta_{0}\right)}^{2}\right)}$$

where the first term is the harmonic potential, and the second term is the nonlinear Lorentzian attractive potential. The assumed angular potential is shown in Figure 9 [43]. It should be noted that the parameters k/I, β/I, ζ, and λ/I are fitting parameters [43]. The demerit of an ODE model is that there are occasionally free-fitting parameters. In other words, an ODE model needs to be validated by comparison with the experimental data or the results of first principles calculations [24].

**Figure 10:**
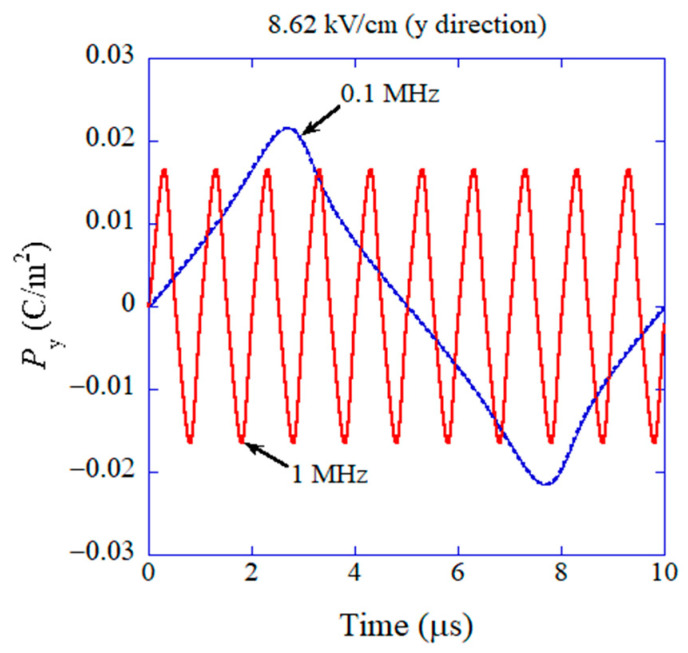
The result of the numerical simulation of the physical ODE model for flexoelectric polarization initially perpendicular to the applied alternating electric field (y-direction) on the component of polarization parallel to the applied electric field (y-direction) as a function of time. Reprinted with permission from ref. [43]. Copyright 2022, MDPI.

**Figure 11:**
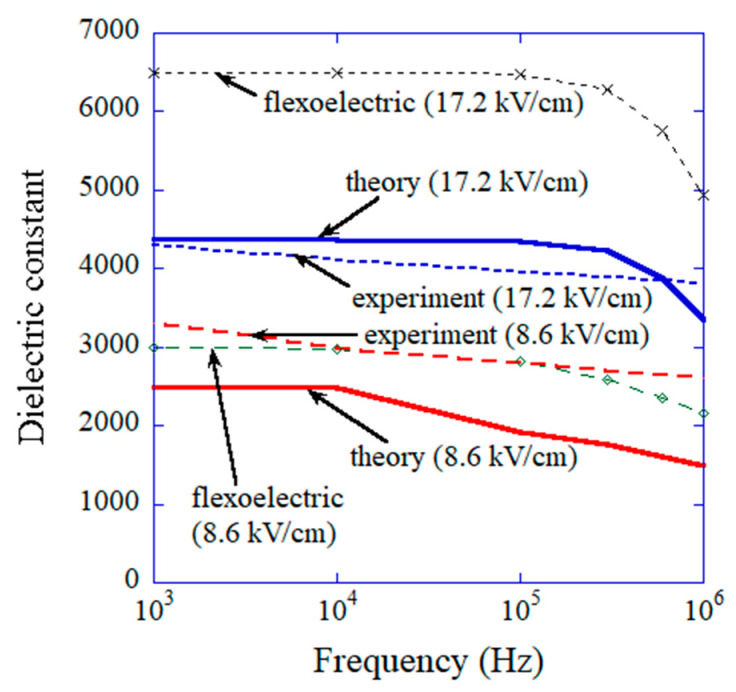
Comparison between the experimental data and the results of the numerical simulations of the physical ODE model on the dielectric constant (due to flexoelectric and ferroelectric contributions) as a function of frequency for the amplitudes of the applied alternating electric field of 17.24 kV cm^−1^ and 8.62 kV cm^−1^. The numerical results calculated solely by flexoelectric polarization are also shown. Reprinted with permission from ref. [43]. Copyright 2022, MDPI.

The results of the numerical simulations obtained via the ODE model are shown in Figure 10 on $P_{y}$ as a function of time [43]. For lower frequency of the applied AC electric field (0.1 MHz), the amplitude of the temporal variation of $P_{y}$ is larger than that at higher frequency (1 MHz) because the damping of oscillation is less due to smaller angular velocity (dθ/dt) compared to the case of higher frequency and the Lorentzian attractive force is weaker at a larger angle ($\left|\theta -\theta_{0}\right|$) as seen in Figure 9 [43]. It results in a higher dielectric constant for lower frequency as shown in Figure 11 [43]. Furthermore, the dielectric constant is higher for a higher amplitude of the applied AC electric field as shown in Figure 11 because the restoring force is weaker for a larger angle ($\left|\theta -\theta_{0}\right|$), and accordingly the amplitude of oscillation of $P_{y}$ becomes much larger [43]. In this way, the mechanism of the results is easily traced in an ODE model, which is one of the merits of ODE modelling [24]. However, there is some discrepancy between the calculated dielectric constant and the experimental data as a function of frequency as shown in Figure 11, although they qualitatively agree, which is one of the demerits of ODE modelling compared to ML. With regard to the computational time of ODE model calculations, it ranges from a few minutes to a few hours, which is almost comparable to that for ML.

## 6. Dielectric Constant

**Figure 12:**
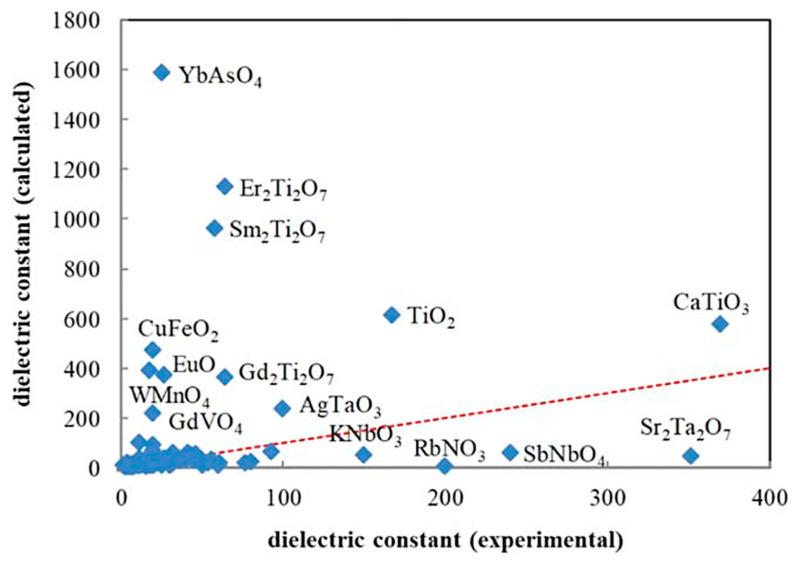
Comparison between the experimental data and the results of the first principles calculations (density functional perturbation theory) on the dielectric constant for 105 selected compounds. The red dashed line from the origin has a unit slope. Reprinted with permission from ref. [9]. Copyright 2019, IOP Publishing, Ltd., Bristol, UK.

In the present section, the prediction of dielectric constants using a combination of first principles calculations and ML reported by Umeda et al. [9] is discussed. As there is a considerable discrepancy between the results of first principles calculations and experimental data on the dielectric constant (Figure 12), a ML model is employed to correct the results of the first principles calculations by training with the experimental data [9]. In the first principles calculations of dielectric constants, density-functional perturbation theory is employed [162,163,164]. The dielectric constant is the sum of the contributions due to ionic and electronic polarizations [9,164]. The dielectric constants given by the first principles calculations shown by the vertical axis of Figure 12 correspond to those for polycrystals given by a simple average of eigenvalues of the single-crystal dielectric tensor [9,164]. In Figure 12, large deviations between the experimental and the calculated values are seen for approximately 20% of the compounds [9]. For compounds containing rare earth elements, the calculated dielectric constants were overestimated. On the other hand, for compounds containing niobium, tantalum, and alkali metal elements, the calculated values were underestimated [9]. The difference between the calculated and experimental dielectric constants depends on the kind of chemical element contained in the compounds. Accordingly, Umeda et al. [9] employed ML to correct the calculated dielectric constants.

**Figure 13:**
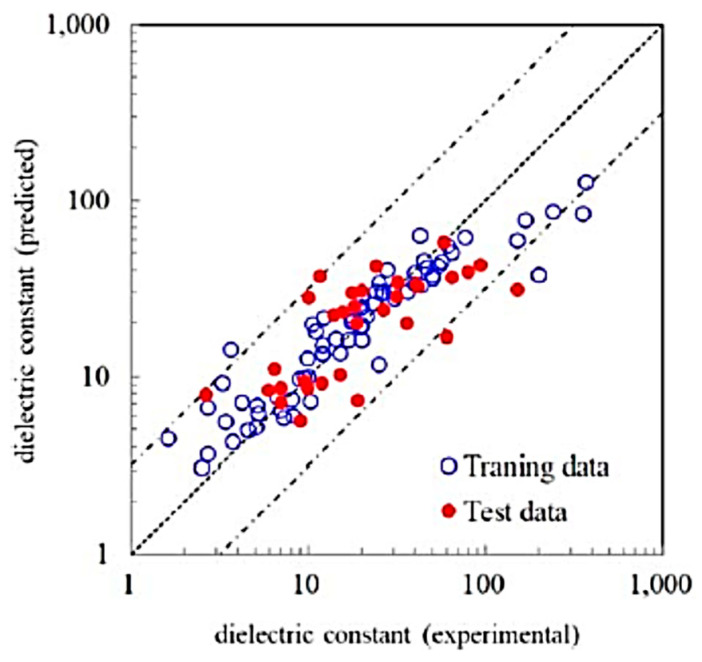
Comparison between the experimental and the ML predicted dielectric constants. The ML prediction was performed using the random forest (RF) regression model with 68 feature variables. The dashed line has a unit slope. The dashed–dotted lines are the upper and lower bounds of the predicted value with 50% error of the logarithmic of the dielectric constant. Reprinted with permission from ref. [9]. Copyright 2019, IOP Publishing Ltd.

**Figure 14:**
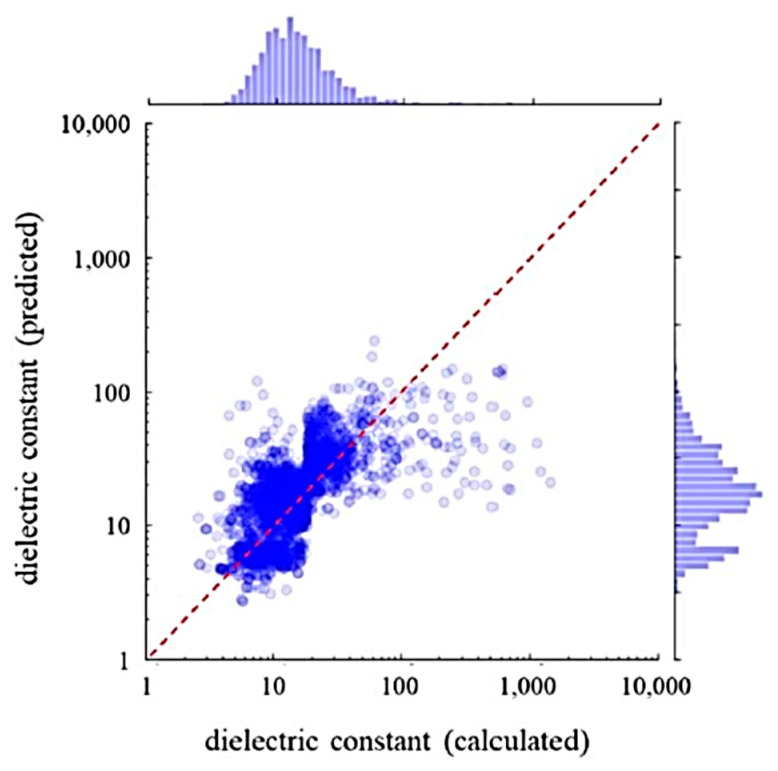
Comparison between the dielectric constants calculated with first principles and those predicted by ML. The ML prediction was performed using the random forest regression model with 68 feature variables. The dashed line has a unit slope. Reprinted with permission from ref. [9]. Copyright 2019, IOP Publishing Ltd.

In Figure 13, the dielectric constants predicted by an RF model and the experimental data are compared [9]. An RF model was trained with the experimental data with 68 feature variables (descriptors) such as the dielectric constant by first principles calculations, ionization potentials, electron affinity, etc. [9]. The significantly important feature variable was the dielectric constant predicted by first principles calculation. Approximately 90% of the test data were within the 50% error range. In Figure 14, dielectric constants predicted by an RF model for 2504 compounds are compared with those predicted by first principles calculations [9]. There is some systematic error in dielectric constants predicted by first principles calculations originating in some elemental features. In conclusion, many dielectric constants predicted by first principles calculations deviate significantly from experimental data (Figure 12). The accurate prediction of dielectric constants by ML is possible by correcting the results of first principles calculations by using some elemental feature variables (Figure 13) [9]. In summary, ML could correct the results of first principles calculations to fit the experimental data.

## 7. Ionic Conductivity in Solid Electrolytes

**Figure 15:**
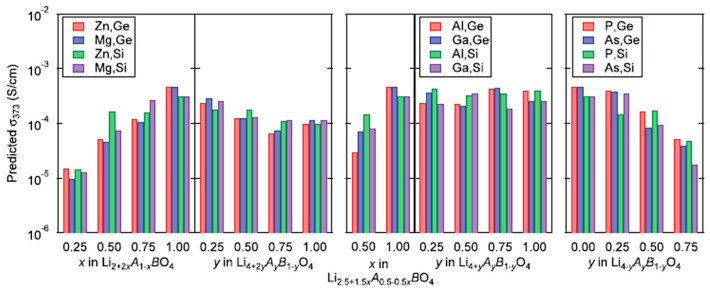
ML-predicted ionic conductivities at 373 K, $\sigma_{373}$, for 72 compositions in the system ${Li}_{8-c}A_{a}B_{b}O_{4}$, where $A^{m+}=Zn,Mg,Al,Ga,P\;or\;As$, and $B^{n+}=Ge\;or\;Si$, and c=ma+nb. The ML prediction was undertaken by using the support-vector regression method with a Gaussian kernel after training with the experimental data of ionic conductivities for a given diffusion coefficient at 1600 K, $D_{1600}$, phase transition temperature, $T_{c}$, and average volume of disordered structure, $V_{dis}$, calculated by first-principles molecular-dynamics simulations. Reprinted with permission from ref. [67]. Copyright 2013, Wiley.

ML was utilized to accelerate the discovery of new solid electrolytes with high ionic conductivity for the application to all-solid-state batteries [10,13,67,165,166,167,168,169,170,171]. Fujimura et al. [67] reported that a ML model could predict ionic conductivity at 373 K for various compositions in the system ${Li}_{8-c}A_{a}B_{b}O_{4}$, where $A^{m+}=Zn,Mg,Al,Ga,P$ or As, and $B^{n+}=Ge$ or Si, and c=ma+nb. The feature variables (descriptors) were the diffusion coefficient at 1600 K calculated by first-principles molecular-dynamics simulations, experimental temperature, the phase transition temperature and the average volume of disordered structures which were obtained by DFT-based calculations [67]. The ML model employed was the support-vector regression (SVR) method. The predicted ionic conductivities at 373 K for various compositions are shown in Figure 15 [67]. The predicted ionic conductivities range from about $10^{-5}$ S ${cm}^{-1}$ to about $10^{-3}$ S ${cm}^{-1}$. However, it can be pointed out that all-dislocation-ceramics [58], which could be a new type of solid electrolyte with high ionic conductivity without dendrite formation filled with appropriate dislocations, could not be predicted by ML because ML could not predict beyond the parameter space of the training (experimental and theoretical) data [10,13,67,165,166,167,168,169,170,171].

**Figure 16:**
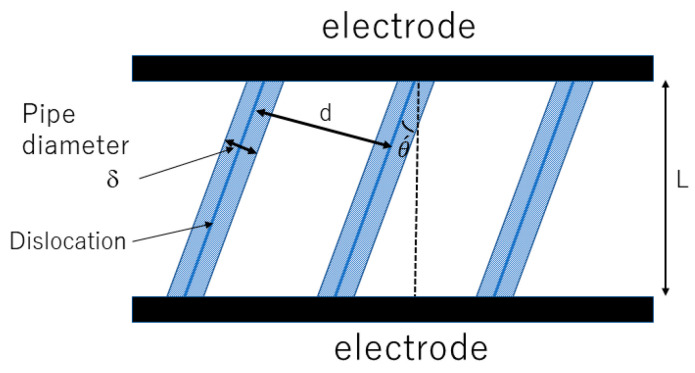
Model of single-crystal solid electrolyte with parallel straight dislocations. Reprinted with permission from ref. [57]. Copyright 2023, IOP Publishing Ltd.

In the following section, how all-dislocation-ceramics was theoretically discovered is discussed [57,58,172]. In the theoretical analysis, parallel straight dislocations in a single-crystal solid electrolyte are considered (Figure 16) [57]. For each straight dislocation, a dislocation pipe is considered along which ionic conductivity is significantly higher than that in the bulk because the concentration of vacancies is considerably higher [59,60,61,62]. The typical diameter of a dislocation pipe is denoted by δ, and the distance between neighboring dislocations is denoted by d which is given by Equation (31) [57]:

(31) $$d=\frac{1}{\sqrt{n_{d}}}$$

where $n_{d}$ is the number density of dislocations. As the ionic conductivity along a dislocation pipe could be several orders of magnitude higher than that in the bulk, dendrites would be generated along dislocation pipes due to the concentration of the ionic current [173]. This could be a severe problem in all-solid-state batteries because of short-circuiting [174]. However, if the electrode surface is completely covered with parallel dislocations, dendrite formation could be avoided because the spatial variation of ionic current density becomes relatively small [57]. This is the condition for all-dislocation-ceramics, which is roughly given by Equation (32):

(32) $$\delta \geq \sqrt{2}d$$

The angle of a straight dislocation relative to the vertical direction to the electrode is denoted by $\overset{´}{\theta }$ (Figure 16). Then, the mean ionic conductivity (σ) of this system is approximately derived as follows [57]:

When δ<d,

(33) $$\sigma =\sigma_{d}{\left(\cos\overset{´}{\theta }\right)}^{2}n_{d}S_{d}+\sigma_{b}{\left(\sin\overset{´}{\theta }\right)}^{2}n_{d}S_{d}+\sigma_{b}\left(1-n_{d}S_{d}\right)-\overset{´}{A}\left(1-n_{d}S_{d}\right)$$

When $\delta \geq \delta_{c}$,

(34) $$\sigma =\sigma_{d}{\left(\cos\overset{´}{\theta }\right)}^{2}+\sigma_{d⊥}{\left(\sin\overset{´}{\theta }\right)}^{2}$$

where $\sigma_{d}$ and $\sigma_{b}$ is ionic conductivity along a dislocation and in the bulk, respectively, $S_{d}$ is the cross-section of a dislocation pipe, $\overset{´}{A}$ is the amplitude of reduction in ionic conductivity by crossing dislocations, and $\delta_{c}$ is the critical diameter of a dislocation pipe for all-dislocation-ceramics given as follows [57]:

(35) $$\delta_{c}=\sqrt{\frac{2}{n_{d}}}$$

In Equation (33), the first term on the right side is the contribution of ionic conduction along dislocations. The second term is the contribution of ionic conduction leaking from dislocations. The third term is the contribution of ionic conduction in the bulk. The last term is the reduction of ionic conductivity by crossing dislocations. In Equation (34), the first term is the contribution of ionic conduction along dislocations, and the second term is that of crossing dislocations.

The spatial variation of ionic current density on the opposite electrode is estimated by the following equation [57]:

When δ<d,

(36) $$j_{dis}=\sigma_{d}\overset{´}{E}{\left(\cos\overset{´}{\theta }\right)}^{2}$$

(37) $$j_{other}=\sigma_{b}\overset{´}{E}+\sigma_{b}\overset{´}{E}{\left(\sin\overset{´}{\theta }\right)}^{2}\left(\frac{n_{d}S_{d}}{1-n_{d}S_{d}}\right)-\overset{´}{A}\overset{´}{E}$$

where $j_{dis}$ and $j_{other}$ is the ionic current density on a dislocation and that on the other area, respectively, on the opposite electrode. When $\delta \geq \delta_{c}$, all the area is completely covered with dislocations and the spatial uniformity of the ionic current density ($\frac{j_{dis}}{j_{other}}=1$) is assumed. Under this condition, dendrite formation may be avoided.

**Figure 17:**
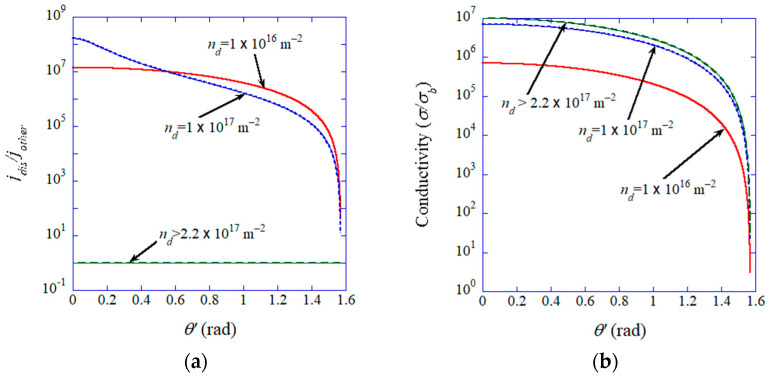
Influence of the dislocation density ($n_{d}$). (**a**) $j_{dis}/j_{other}$, where $j_{dis}$ and $j_{other}$ are the ionic currents on a dislocation and on the other area, respectively, on the opposite electrode, as a function of the angle ($\theta^{\prime }$) of the parallel dislocations. (**b**) The mean ionic conductivity ($\sigma /\sigma_{b}$) where $\sigma_{b}$ is ionic conductivity in the bulk. Reprinted with permission from ref. [57]. Copyright 2023, IOP Publishing Ltd.

The numerical results of Equations (33), (34), (36), and (37) are shown in Figure 17 [57]. The assumed parameters are δ=3 nm [175], and $\frac{\sigma_{d}}{\sigma_{b}}=10^{7}$ [61,176], $\frac{\sigma_{d⊥}}{\sigma_{b}}=10^{-2}$. When the dislocation pipe diameter is δ=3 nm, the condition for all-dislocation-ceramics is $n_{d}>2.2\times 10^{17}$ $m^{-2}$. Under the condition of all-dislocation-ceramics, the ionic current density is spatially uniform and the mean ionic conductivity could be seven orders of magnitude larger than that in the bulk (Figure 17) [57]. Below the critical dislocation density, on the other hand, dendrite formation may be inevitable because the ionic current is concentrated along dislocations (Figure 17) [57].

**Figure 18:**
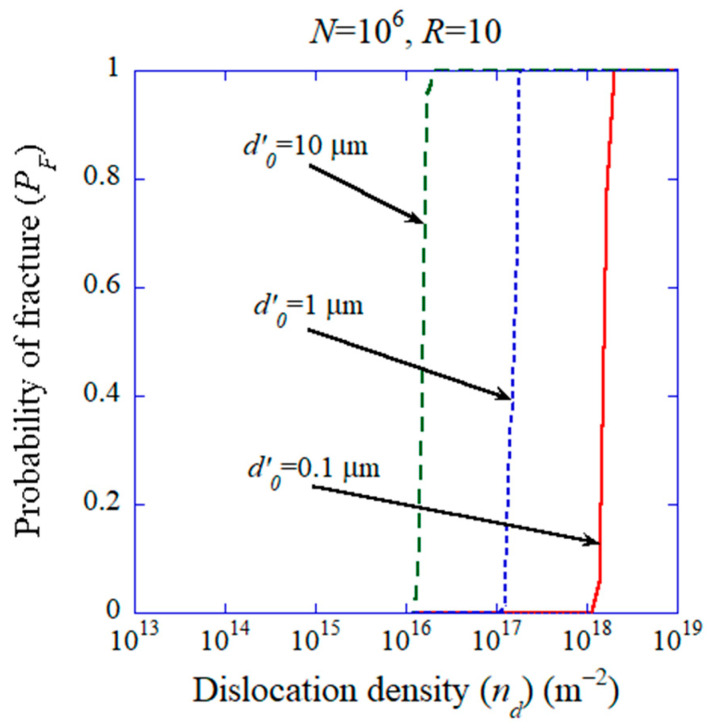
The results of numerical calculations for the probability of fracture ($P_{F}$) as a function of dislocation density when the number of microcracks is $N=10^{6}$ for various values of ${d^{\prime }}_{0}$ which characterizes the size distribution of pre-existing microcracks. Reprinted with permission from ref. [172]. Copyright 2023, IOP Publishing Ltd.

Recently, it has been suggested that the introduction of dislocations is useful for improving the functional, electrical and mechanical properties of ceramics, which is called dislocation engineering [62,63,64,65,177,178,179,180]. In many cases, dislocations are introduced into ceramics by applying compressive stress at room temperature or elevated temperatures [62,177,178,179,181,182,183,184]. Thus, whether high dislocation density such as $2.2\times 10^{17}$ $m^{-2}$ or more is achievable without the failure of a specimen becomes an issue. In order to study it theoretically, the probability of fracture $P_{F}$ in volume $\overset{´}{V}$ of a specimen given by Equation (38) is numerically calculated as shown in Figure 18 [172]. Due to the statistical nature of the number and size of pre-existing microcracks in a specimen, the occurrence of fracture is expressed by a probability [172]:

(38) $$P_{F}=1-exp\left[-\overset{´}{n}\overset{´}{V}exp\left(-{\left(\frac{\overset{´}{d_{c}}}{\overset{´}{d_{0}}}\right)}^{\overset{´}{\overset{´}{m}}}\right)\right]$$

where $\overset{´}{n}$ is the number concentration of pre-existing microcracks, $\overset{´}{d_{c}}$ is the critical diameter of a microcrack for fracture derived by the Griffith criterion, and $\overset{´}{d_{0}}$ and $\overset{´}{m}$ are constants which characterize the size distribution of microcracks [172]. From the numerical results shown in Figure 18, a dislocation density higher than $2.2\times 10^{17}$ $m^{-2}$ is achievable if the characteristic size $\overset{´}{d_{0}}$ of pre-existing microcracks in a specimen is smaller than about 1 μm [172]. In Figure 18, R=10 is assumed, where R is defined as the ratio of compressive strength to tensile strength [172].

In conclusion, from the physical algebraic equations (Equations (33)–(37)), all-dislocation-ceramics is theoretically discovered, although experimentally it has not yet been achieved. In other words, discussions based on physical algebraic equations could lead to prediction of all-dislocation-ceramics, which could not be predicted by ML because this is beyond the parameter space of pre-existing experimental data. If there are already experimental data of ionic conductivity as a function of dislocation density, it may be possible for all-dislocation-ceramics to be predicted by ML. However, even in this case, consideration by human researchers on the importance of dislocation density is needed beforehand. In future, however, generative pre-trained AI may possibly overcome the difficulty and predict unknown optimal conditions for solid electrolytes beyond the pre-existing parameter space of the experimental data [185,186,187]. For example, generative AI pre-trained with many literatures on ionic conductivity could point out the importance of dislocation density and could predict all-dislocation-ceramics even without referring to the papers of all-dislocation-ceramics. Nevertheless, the final decision as to whether the prediction made by AI is seriously considered should be made by human researchers.

## 8. Merits and Demerits of ML

In Table 1, ML, first principles calculations, and physical or phenomenological ODE model calculations are compared with respect to interpretability, the accuracy of predicted values (predictive power), the number of fitting parameters, the amount of required experimental data, computational cost, and the need for model validation. As already noted, there are variety of ML models from a white-box to a black-box model [69]. Recently, some attempts have been made to improve the interpretability of ML models, as already noted [2,8,69,79,80,81]. However, some caution toward the interpretability of ML models is needed because sometimes non-physical spurious correlation results from ML models [188]. Some correlations derived by a ML model need to be evaluated by human researchers considering their physical or chemical meaning. Accordingly, the interpretability of ML models is relatively low compared to first principles calculations and ODE model calculations. For first principles calculations, it is basically possible to see how some mechanism works for the calculated results. However, it is sometimes more difficult to interpret the calculated results due to the more complex nature of the first principles calculations compared to ODE model calculations. Thus, it is generally the case that the interpretability of ODE model calculations is higher than that of first principles calculations [24].

As ML is basically a fitting procedure of experimental data as a function of descriptors, the accuracy of the predicted values (predictive power) could be relatively high if excellent descriptors are found and the training experimental data contain negligible error. However, outside the range of the training (experimental) data, the predictive power could drop off. Furthermore, as discussed before in relation to all-dislocation-ceramics, ML could not predict experimental results beyond the parameter space of the training data. As there was no training data of ionic conductivity as a function of dislocation density, ML could not find a possible new kind of solid electrolytes, namely all-dislocation-ceramics. On the other hand, from physical algebraic equations, all-dislocation-ceramics could be found, as already discussed. In summary, physical or chemical consideration by human researchers is important to predict experimental results beyond the parameter space of pre-existing experimental data. Even if generative pre-trained AI could overcome the difficulty of ML in future, the final decision as to whether the prediction made by AI is seriously considered should be made by human researchers.

The predictive power of first principles calculations could be reasonably high for some cases. However, for other cases, it could not be so high because of approximations used in the exchange–correlation potential and neglect of defects and impurities, etc. As already discussed, ML could correct the results of first principles calculations by training with the experimental data [9]. With regard to ODE model calculations, the predictive power is generally not high because an ODE model is not fully based on the first principles. ODE models are usually useful for qualitative or semi-quantitative discussion of some mechanism for experimental results [24]. Thus, for an ODE model, the validation of the model by comparing the calculated results with the experimental data or the results of first principles calculations is required [24]. Nevertheless, in some cases, an ODE model could predict experimental results excellently, as in the case of sonochemistry and sonoluminescence [148,149,154].

For ML, the number of fitting parameters could be relatively small for linear regression, but it could be very large for deep learning (DL). For first principles calculations, for many cases, the number of fitting parameters is relatively small (or possibly none). However, in chemistry, it is sometimes large when exchange–correlation functionals are fitted to databases [92,189]. For ODE model calculations, the number of fitting parameters could be small (or possibly none) [148,149,154], but for other cases, there are several free-fitting parameters [33,43].

One of the limitations of ML is that the required amount of pre-existing experimental data for training of a model is very large. In other words, if there are not enough experimental data, ML cannot be used to predict experimental results. On the other hand, first principles calculations do not necessarily require pre-existing experimental data. For ODE model calculations, some coefficients could be derived from some experimental data, but for other cases, pre-existing experimental data are not required.

With regard to computational cost, it includes computational time, required memory size and hard-disk capacity, cost of coding, etc. In general, first principles calculations need high computational cost. On the other hand, ML needs much less computational cost for many cases. ODE model calculations are usually computationally more economical [24].

For any computational model including the model of first principles calculations, validation of a model by comparing the calculated results with experimental data is required [93]. In particular, a ML model and an ODE model need validation because they are not fully based on first principles.

Finally, a recently developed ML model that analytical equations can be automatically derived from experimental data is briefly discussed [190,191,192]. By randomly selecting analytical equations in a computer, some formula is derived from experimental data, which fits the experimental data in a most excellent manner. In such a ML model, some physical law could be automatically derived from experimental data, and interpretability could become relatively high [190,191,192]. In 1601, Johannes Kepler started the analysis of the observed data on trajectories of planets [190]. After 4 years and about 40 failed attempts to fit the Mars data to various ovoid-shaped orbits, he discovered that Mars’ orbit was an ellipse with the Sun being one of the focal points, which is Kepler’s first law [190]. The above ML model has a possibility to automatically accelerate such analysis of experimental data to derive analytical equations of natural laws in future.

## 9. Conclusions

The merits and demerits of machine learning (ML) are discussed, compared with first principles calculations and physical or phenomenological ODE model calculations. ML is basically a fitting procedure of experimental data as a function of descriptors. If excellent descriptors can be found and the training data contain negligible error, the predictive power of a ML model could be relatively high. However, a ML model hardly predicts experimental results beyond the parameter space of the training (experimental) data. On the other hand, numerical calculations of physical algebraic equations as well as ODE model calculations could predict experimental results beyond the parameter space of pre-existing experimental data. One example is all-dislocation-ceramics, which is a possible new kind of solid electrolyte filled with appropriate dislocations for high ionic conductivity without dendrite formation.

## Figures and Tables

**Table 1:** Comparison between machine learning (ML), first principles calculations, and physical or phenomenological ODE model calculations.

	Machine Learning	First Principles	ODE Model
Interpretability	Low~Medium	Medium~High	High
Accuracy of predicted values	Medium~High (Low outside the range)	Medium~High	Low~Medium
Number of fitting parameters	Small~Very Large	None~Medium	Small~Medium
Amount of required experimental data	Very Large	None~Small	Small~Medium
Computational cost	Low~Medium	High	Low~Medium
Model validation	Strongly Required	Required	Strongly Required

## Data Availability

Data are contained within the article.
